# A machine learning-based classification model to support university students with dyslexia with personalized tools and strategies

**DOI:** 10.1038/s41598-023-50879-7

**Published:** 2024-01-02

**Authors:** Andrea Zingoni, Juri Taborri, Giuseppe Calabrò

**Affiliations:** Department of Economics, Engineering, Business and Society, University of Tuscia, 01100 Viterbo, Italy

**Keywords:** Neurodevelopmental disorders, Electrical and electronic engineering

## Abstract

Dyslexia is a specific learning disorder that causes issues related to reading, which affects around 10% of the worldwide population. This can compromise comprehension and memorization skills, and result in anxiety and lack of self-esteem, if no support is provided. Moreover, this support should be highly personalized, to be actually helpful. In this paper, a model to classify the most useful methodologies to support students with dyslexia has been created, with a focus on university alumni. The prediction algorithm is based on supervised machine learning techniques; starting from the issues that dyslexic students experience during their career, it is capable of suggesting customized support digital tools and learning strategies for each of them. The algorithm was trained and tested on data acquired through a self-evaluation questionnaire, which was designed and then spread to more than 1200 university students. It allowed 17 useful tools and 22 useful strategies to be detected. The results of the testing showed an average prediction accuracy higher than 90%, which rises to 94% by renouncing to guess the less-predictable 8 tools/strategies. In the light of this, it is possible to state that the implemented algorithm can achieve the set goal and, thus, reduce the gap between dyslexic and non-dyslexic students. This achievement paves the way for a new modality of facing the problem of dyslexia by university institutions, which aims at modifying teaching activities toward students’ needs, instead of simply reducing their study load or duties. This complies with the definition and the aims of inclusivity.

## Introduction

Specific learning disorders (SLDs) represent a set of neurodevelopmental impairments that cause substantial difficulties in learning skills, ranging from reading to writing, as well arithmetical issues^1^. Thus, students showing SLDs often face deep issues that affect their career in terms of both results and arising of psychological consequences^2^. Even though several studies suggest that people having a deficit in one learning domain frequently show deficits also in other domains^3^, three main independent categories of SLD can be distinguished, based on the impaired learning skills. These are: (i) dyslexia, when issues related to reading are predominant; (ii) dysgraphia, when the issues mostly concern writing, and (iii) dyscalculia, when arithmetical skills are compromised.

Recent studies demonstrate that up to 20% of the worldwide population may have a SLD and that dyslexia is the most common among them^4^. Despite this, the phenomenon has gained the deserved attention only in the last decade. As an example, almost 7000 scientific papers, indexed in Web of Science, addressed the topic of dyslexia since 2013, and approximately the same number addressed it in the previous 20 years, namely in a double time span. Although, as mentioned above, dyslexia is associated with issues related to reading, as a direct consequence affected people also experience difficulties in comprehension and memorization of concepts, as well as in taking notes during lessons^5^. It is thus clear that specific and effective support interventions are needed to ensure equal opportunities for dyslexic students.

Since it has been demonstrated that early recognition of dyslexia can have a significant impact on limiting the issues it causes^6^, diagnostic tools are of paramount importance. There is a relative agreement on the dyslexia diagnosis methodologies used in clinical and research fields^7^. Generally, clinical practice consists in the administration of standardized tests to quantify reading ability, together with the analysis of intellectual aspects; if the former is lacking but no problems are detected in the latter (for the Italian context, it means an intelligence quotient—IQ—equal or greater to 85), the presence of dyslexia is declared. In the last decades, the advent and the constant progress in biomedical engineering have raised the possibility to propose innovative approaches. Firstly, the importance of analyzing neurological data have been revealed, encouraging the use of functional magnetic resonance imaging (fMRI) to identify anomalies in the cerebral morphology of dyslexic subjects^8^. Further, similar approaches have been proposed by evaluating the activation patterns gathered from electroencephalogram (EEG) tests and, in particular, the spectral features obtained while analyzing different brain areas^9^.

Significant advances have been made not only in diagnosis instrumentation but also in diagnosis techniques, mainly thanks to artificial intelligence (AI), which has offered interesting new possibilities to analyze data, so much that it can be now considered as the effective turning point with respect to the most common practices. For example, machine learning (ML) algorithms were used to generate automatic predictions of the presence of dyslexia based on tests’ results, as in^10^, where an artificial neural network (ANN), fed with the outcomes of the Gibson test, was capable to identify dyslexic subjects with an accuracy close to 90%. Similar outcomes were found in^11^ and^12^. The former showed that a support-vector machine (SVM) algorithm is able to discriminate reading disorders again with a percentage of success of about 90%. The latter, instead, suggested how a fuzzy algorithm can help psychologists to detect potential cases of dyslexia. A human-driven machine learning algorithm was proposed in^13^, where the prediction of children at risk for dyslexia was detected with an accuracy of 99%. In addition, a screening tool, named as DysLexML, based on the combination of data gathered from eye movements during text reading and on the application of SVM was implemented and associated with an accuracy of 97%^14^. It is worth noting that, if on the one hand several efforts have been made to improve dyslexia diagnosis on children, on the other hand specific tools to detect and/or monitor dyslexia in adult subjects are still missing, with the exceptions of LSC-SUA^15^ and Adult Dyslexia Battery^16^ tests that, however, do not exploit at all the potential offered by information technology (IT).

Unfortunately, when early diagnosis fails or is not performed, the issues caused by dyslexia during the learning process tend to be more severe and the probability to solve, or at least mitigate them, decreases consistently^6^. In this case, developing specific support tools and strategies become of paramount importance to provide help properly and usefully. Again, IT and, in particular, AI can offer a wide variety of promising solutions. One of the most interesting was presented in^17^, where an assistive reading tool was designed by combining read-aloud technologies and AI paradigms applied to eye tracking. A pilot study on 20 children, ranged from 8 to 10 years old, showed an increase of 24% of a text comprehension score. Even in^18^, an assistive digital platform was implemented in Malay language. Hidden Markov models and an ANN were used to make the platform self-adaptable to the learning environment. Another digital support tool, called ALEXZA, was introduced in^19^. It helps young dyslexic students while reading, by using AI to recognize text from pictures and read it aloud, also suggesting common synonyms in case of unfamiliar words. A further online platform for e-learning has been proposed by^20^ and tested on students ranged from 8 to 12 years old. This platform is able to adapt the methodology for providing the correct learning approach based on user profile and progress. An interesting approach that explores an alternative way to offer support to dyslexic students was introduced in^21^. Here, AI was employed to develop an augmentative and alternative communication (AAC) model, capable of classifying questions uniquely and to provide users with related pictograms. The study reported a decrease up to more than 66% in the effort and time to interact among the users. Finally, the work^22^ proposed an adaptive e-learning method able to detect the dyslexia type and to offer appropriate learning methodology to the user. However, after the first identification, no further adaptations were applied leading to a low possibility to customize the methodology based on user needs and progress. From this overview, it appears clearly how several efforts have been made to help students with dyslexia, by offering adaptive e-learning methods, but such efforts are totally addressed to students at primary schools (7–12 years old). On the contrary, no digital platform based on AI have been proposed for university career, even though it is well known that the inclusion of student with dyslexia in higher education is one of the open challenges nowadays. An exception to this is represented by^23^. It describes the project VRAIlexia, in which AI is employed, jointly with virtual reality (VR)^24^, to develop a platform capable to offer personalized support to dyslexic students during their academic career.

The work here presented is framed within this project. In particular, it is aimed at building a classification model of the most useful digital tools and learning strategies, customized for each university student with dyslexia, Based on the challenges they have encountered during their educational journey. The goal is to provide tailored support methodologies to each student, in order to fill the gap between dyslexics and non-dyslexics, which very often arise in the years of university. ML techniques have been explored and they proved to be an optimal tool to achieve the purpose. In the next section, the used methodology is presented in detail, focusing on all the main aspects, from data collection and processing to algorithms choice, training and testing. Then, in the “Results” section, these are shown and discussed. The final section is left to the conclusions.

## Research methodology

### Data collection

To gather the data on which to train and then test the final ML prediction algorithm to be implemented, a questionnaire was elaborated. It is divided into three main sections. The first one concerns aspects related to demography (age, gender, provenience, etc.) and dyslexia history (presence of relatives with dyslexia, possible problems during study career, received support, etc.), and will not be taken into account in this work. However, a wide analysis of its information has been made in^25^. The second section is composed by questions related to the issues that students may have experienced during their learning path. The third section, instead, contains questions about the supporting tools and strategies (or services) they have found most useful to mitigate learning problems. In Tables 1 and 2 (a) and (b), the complete list of asked questions in the sections of interest is reported. In the second section, the participants to the questionnaire were asked to express their feeling about how severely they have been affected by each one of the listed issues, by choosing an option among: “*not at all*”, “*very little*”, “*little*”, “*medium*”, “*much*” and “*very much*”. In the third section, instead, the participants could express their opinion about the usefulness of each of the present supporting tools and strategies, with the same options as above but with the addition of “*never tried*” and “*don’t know*” for the answers related to the tools. This allows a discrimination between a useless tool and an unknown one, so as not to bias the results. In both cases, an empty textbox was inserted, where the participants could insert possible additional information.

**Table 1:** List of the questions about the possible issues experienced by dyslexic students during their career, asked in the 2nd section of the questionnaire.

Id	Have you ever experienced the following issues?
I1	Reading difficulties
I2	Text comprehension difficulties
I3	Uncommon words understanding
I4	Lessons comprehension
I5	Concentration difficulty while studying
I6	Concentration difficulty during in-class lessons
I7	Concentration difficulty during online lessons
I8	Verbal short-term memory impairment
I9	Verbal long-term memory impairment (memory loss during exams)
I10	Study scheduling
I11	Note-taking difficulties
I12	Lack of time to prepare exams

**Table 2:** List of the questions about the most useful tools (a) and strategies (b) for dyslexic students, asked in the 3rd section of the questionnaire.

(a)		(b)	
Id	Have you considered the following supporting tools as useful?	Id	Have you considered the following supporting strategies as useful?
T1	Audiobook with human voice	S1	Someone that read study material
T2	Audiobook with artificial voice	S2	Making my own concept maps
T3	Words in different colors	S3	Making my own schemes
T4	Specific font for dyslexic	S4	Making my own summaries
T5	Use of smart pen or tablet to take notes and record voice	S5	Repeating studied material
T6	Clear layout of the study material	S6	Highlighting keywords by my own
T7	Highlighted keywords	S7	Underlining words with different colors
T8	Digital concept maps	S8	Study groups
T9	Digital schemes	S9	Tutor
T10	Summaries	S10	Participating or creating students’ associations to exchange information
T11	E-book	S11	In-class lessons
T12	Digital tutor	S12	On-line lessons availability
T13	Use of images for words memorization and understanding	S13	Pauses during lessons
T14	Use of images for concepts memorization	S14	Lessons slides availability
T15	Audio recording of the lessons	S15	Recording lessons
T16	Video lessons	S16	Taking notes
T17	Integrating study material using internet	S17	Early availability of courses programme
		S18	Dividing exams in multiple shorter modules
		S19	Only written exams
		S20	Only oral exams
		S21	Taking exams in presence of the sole professor
		S22	Database of study material created by other students

The questionnaire was created by a group of psychologists having a solid knowledge about dyslexia in the adult population. They initially sketched out a first list of items, based on their professional experience. Then, they interviewed a sample of twenty university students with dyslexia to refine such a list. Finally, another group of thirty university students with dyslexia filled the questionnaire and gave a feedback about its clearness and accessibility. Such feedback was taken into account to modify it accordingly.

The optimized version of the questionnaire was then published online and spread to people compliant with the following characteristics:

1. Having a certified diagnosis of dyslexia.
2. Being native Italian speaker. Native speaker in other languages was excluded, since each language has its own peculiar features, which cause problems of different nature to dyslexic students. Thus, it is not a proper approach to consider more than one language jointly^26^.
3. Being more than 18 years old.
4. Attending university or having finished or abandoned it less than five years before the filling of the questionnaire.

As previously mentioned, comorbidity with other SLDs is likely to occur. Since their presence could bias the answers, students that have a certificate of the simultaneous presence of SLDs other than dyslexia have been discarded. Dyscalculia and Dysgraphia will be, singularly, the object of other two similar studies. Handling the three disorders singularly should avoid biases and provide more targeted results.

The collection of the data, as well as all the experimental protocols employed in this research were subjected to a double conformity check. Indeed, they were assessed first by the Ethics Committee of the University of Tuscia and then by the National University Conference of Disability Delegates (CNUD), which is an entity that represents the policy and activities of Italian universities, related to students with SLD and disabilities. The result of the assessment was positive in both cases. In addition, data collection was conducted according to the ethical standards outlined in the 1964 Declaration of Helsinki. The collected data were treated according to the articles 13–14 of the GDPR 2016/679 of the European Union, to ensure the privacy of the participants to be respected. Specifically, data have been taken and processed completely anonymously, and used only for research purpose. All the participants have given their informed consent before filling the questionnaire, by digitally signing an agreement.

### Prediction algorithm design

As anticipated, only the questionary items about the issues encountered by dyslexic students during their career and about the tools and strategies they found more useful to face such problems were taken into account in designing the classification model. In particular, the issues were used as input to train and then to test AI algorithms (the predictors), whereas the tools and strategies were used as output (the labels) for each observed sample. This choice, jointly with the nature of the available data, suggested relying on supervised ML techniques^27^. Deep learning algorithms would indeed be likely to result in overfitting, whereas reinforcement learning algorithms would have no sequential data available on which being trained^28^. Furthermore, their higher complexity would not be justified for the addressed problem.

**Figure 1:**
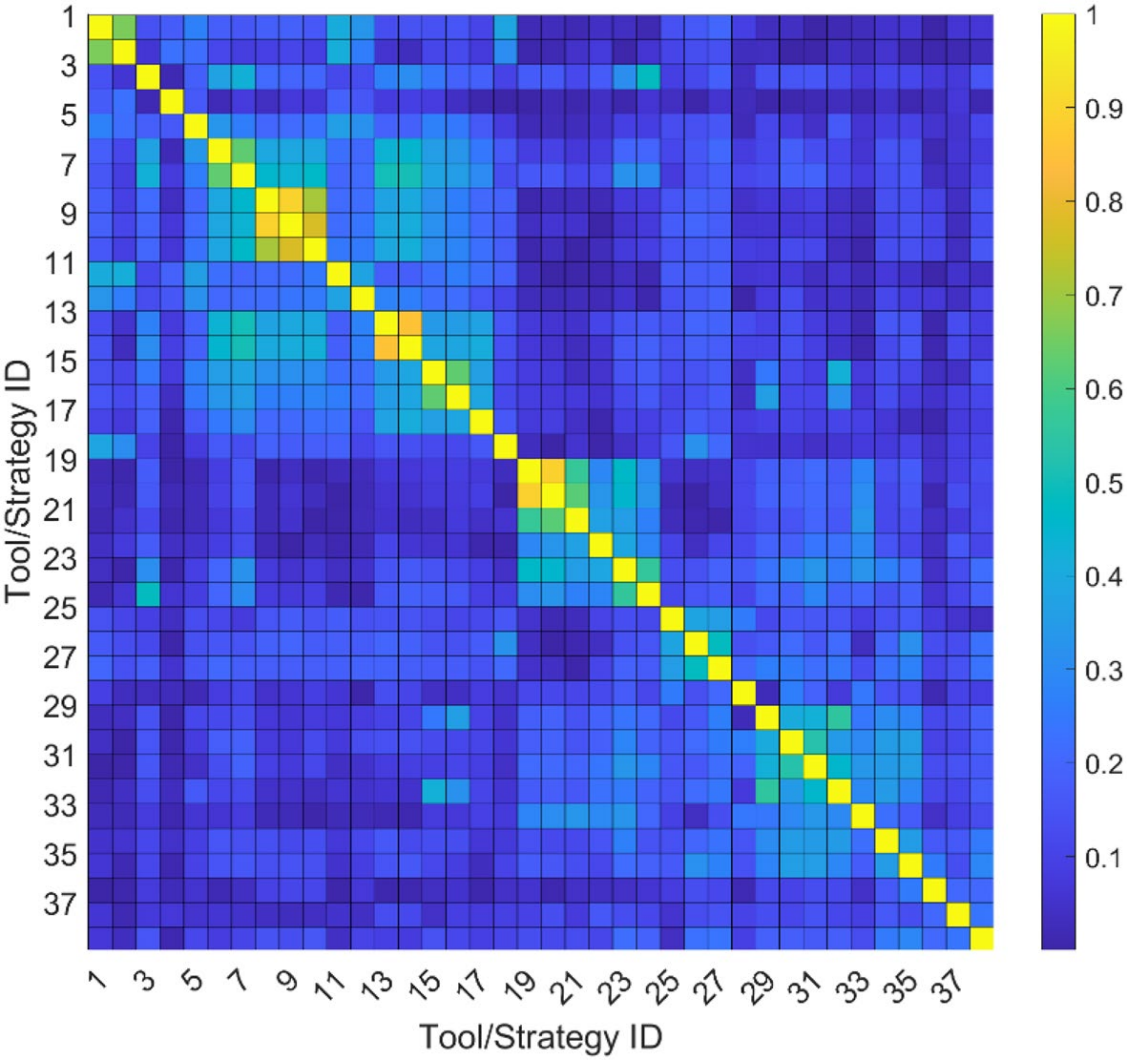
Spearman’s cross-correlation (absolute value) matrix of the scores given (as in Table 3) to the usefulness of tools and strategies. These are indicated with an ID number ranging from 1 to 17 (for the tools) and from 18 to 39 (for the strategies), whereas the correlation values are expressed with colors, whose values are mapped in the color bar on the right.

A preliminary choice that had to be made concerned whether to treat the output variables jointly or singularly and, in the first case, how to group them. The choice depends on if the output variables, or some of them, are considered as correlated to each other or not. Four options are meaningful: (i) all the variables are considered as correlated and, thus, they are treated jointly (in this case, the labels would be vectors containing 39 usefulness scores that were given to the 17 tools and to the 22 strategies); (ii) all the tools and all the strategies are considered as intra-correlated but not inter-correlated, thus the variables are split into two groups (in this case, two predictions would be made, one using a 17 elements vector with the scores given to the tools and the other using a 22 elements vector with the scores given to the strategies, as labels); (iii) following a correlation criterium, some groups of variables are considered as intra-correlated but not inter-correlated thus, they are divided into n groups (in this case, n predictions would be made, each using a vector with the scores given to the tools/strategies within a specific group, as label); (iv) the variables are considered as uncorrelated (in this case a single prediction would be made for each different tool/strategy, using the score given to such a tool/strategy as label). Even if it could be intuitively hypothesized that some of the tools or strategies listed in Table 2 have some kind of correlation, no evidence is present in the literature about support methodologies that correlate to each other. Furthermore, cross-correlation matrix ${\rho }_{X,Y}$ was calculated statistically, by assigning a score to the given answer about the usefulness of each tool/strategy and considering it as the value assumed by that variable, as explained in detail in the next subsection and as shown in Table 3. The chosen correlation criterium was Spearman’s one, since it is particularly suitable for ordinal variables, like the considered ones. Thus:

1 $${\rho }_{X,Y}=1-\frac{6\cdot \sum_{i=1}^{{N}_{oss}}{\left({x}_{i}-{y}_{i}\right)}^{2}}{{N}_{oss}\cdot \left({N}_{oss}^{2}-1\right)} ,$$

where ${x}_{i}$ and ${y}_{j}$ are the $i$-th observation of two generic output variables, namely the scores given to two tools or strategies, and, ${N}_{oss}$ is the number of available observations. Figure 1 shows a graphical representation of ${|\rho }_{X,Y}|$, where the 17 tools has been indicated with the numbers from 1 to 17 and the 22 strategies with the number from 18 to 39, whereas the absolute value of the Spearman’s correlation coefficient that is, each entry of ${|\rho }_{X,Y}|$, has been expressed with colors, whose values are derivable from the color bar. Most of the pairs of variables have a weak correlation. Only 4 of them in 741, namely less than 0.54%, have a strong correlation, stated by ${|\rho }_{X,Y}|>0.7$. Thus, option (iv), namely considering output variables singularly, is the most meaningful and was chosen. This choice also gave the possibility to use a different ML algorithm for the prediction of each variable, improving the overall accuracy. In fact, one of the algorithms could be the strongest in predicting a variable $j$ but weaker than another in predicting variable $k$. Thus, by using only the former, a worse accuracy would be obtained in predicting $k$, whereas by using only the latter, a loss of accuracy would be experienced in predicting $j$. Using the best-predicting algorithm for each variable, instead, led to the best achievable accuracy. The same consideration was applied to the algorithms’ setup: the best setup of an algorithm for the prediction of one of the variables may not be the best to predict another variable. Thus, different setups were chosen for every output variable.

**Table 3:** Correspondence among answers to the questionnaire and assigned score for the algorithms training/testing.

Answers	Equivalent score
Not at all	0
Very little	1
Little	2
Medium	3
Much	4
Very much	5

Four of the best performing supervised ML algorithms for classification^29,30^ were selected to implement the classification model, namely:

- random forest (RF);
- linear/logistic regression (LR);
- k-nearest neighbors (kNN);
- support-vector machines (SVM).

This choice was motivated also by the fact that these algorithms present different classification abilities^31^. Thus, their joint use ensures exhaustivity in the performed research.

Appropriated boosting technique were applied, when recommended. In particular, ADABoost was used with RF and kNN and Gradient Boosting with LR. These techniques were preferred to newest one, as XGBoost, since their complexity does not compensate their performance in dataset as large as the used one, and since ADABoost demonstrated to work better in binary classification problems^32^, as in the considered case (for the size of the dataset and the binarization of the problem, refer to the “Preprocessing of the data” section). No boosting techniques were applied to SVM, since this is already a strong classifier and transform it in a weak one to make it a strong one again with boosting^33^ did not seem a reasonable solution for this work.

It is worth repeating that, for what previously stated, the finally implemented prediction algorithm is not one of the 4 listed above with a particular setup, but a sort of super-algorithm that operate the prediction of each single tool or strategy by relying on the best-performing algorithm within the list, with the most performant setup for that specific tool/strategy.

### Preprocessing of the data

The collected database was composed by 1259 answers to the questionnaire. Among them, 42 were discarded for several reasons, as incompleteness, impossibility of verifying the presence of dyslexia, comorbidities with other SLDs, non-compliance with the four criteria exposed in the “Data collection” section and random filling. The participants that fall into the last categories were detected by the expert psychologists. However, in order not to perturbate the results, they discarded only the very evident cases of random filling as, for example, questionnaires in which the same answer was given to more than 80% of the items, or a questionnaire in which the participant stated that they did not know any of the support tools. The remaining 1217 answers were preprocessed, in order to have a suitable data format to run ML algorithms training and testing. Thus, firstly, a score was assigned to each possible answer to the three groups of questions about encountered difficulties, support tools and support strategies respectively, by following the equivalences shown in Table 3. Concerning the answers “*never tried*” and “*don’t know*” in the support tools questions group, if more than 15% of the participants selected one of this two options for any question, then such a tool was excluded from the analysis. Otherwise, the score was inferred by using the one equivalent to the most frequent answer, namely the mode of the scores given to the considered tool, in order not to decrease the number of samples. Only the tool T4, namely the use of specific fonts for dyslexic students, did not pass the check and was excluded. The open commentaries of the students were also examined, but no significant additional information has been found.

Before starting with the training of the algorithm, a further analysis of the collected data was conducted, to verify their distribution. Concerning the input variables, namely the issues encountered by dyslexic students, a clear prevalence of the scores between 1 and 5 was noticed. This trend is often observed in clinical samples of psychology studies. However, in this case, the presence of 0 scores is considerably lower than in other case. The pool of psychologist that supported the experiment ascribed this to the lack of self-esteem that makes dyslexic students be more pessimistic when they have to self-evaluate their problems^2^. Further analysis will be performed about it. Thus, a switch from a 0-to-5 to a 1-to-5 score was operated, by setting to 1 the few answers with score 0 or 1. After the reset of the scale, the distribution of the scores is approximately uniform for every issue. Concerning the output variables (namely the support tools and strategies) instead, the distribution is imbalanced for some of them, with a maximum ratio, among all, of almost 1/26 between the number of answers with the most given score and the number of answers with the less given one. Since the scope of the algorithm is to indicate if a specific support methodology is useful or not, the scores of the output variables were thresholded, so as to obtain the desired binary response. In particular, scores lower than 2.5 were considered as a statement of uselessness, whereas scores higher than 2.5 were considered as a statement of usefulness. The selection of 2.5 as the threshold stays with the fact that this is the central value of the 0 to 5 score interval, thus ensuring that the same number of possible answers is assigned to the two classes “useful” and “useless”. It is worth noting that, by making this choice, the participant students for which a certain tool/strategy is marked as useless actually answered that it is “not at all”, “very little” or, at most, just “little” useful; conversely, the students for which a certain tool/strategy is marked as useful actually stated that it has a “very much”, a “much” or, at least a “medium” utility, which is reasonable. After the thresholding, the highest imbalance between the (two) classes decreased to a ratio around 1/5, with a prevalence of usefulness statements. To deal with such an imbalance, at the moment of verifying the accuracy of the final algorithm, a different weight will be assigned depending on if the methodology is predicted as useful or as useless, as explained later in detail.

### Training and testing of the ML algorithms

The four ML algorithms that compose the final prediction algorithm were trained and tested multiple times, by using different setups, in order to find the one that allow the highest accuracy to be achieved. As said in the “Preprocessing of the data” section, this process was repeated independently for each support tool or strategy, to increase the overall accuracy of the final prediction algorithm.

Thus, first the dataset has been randomly split into two, by using 75% of it for the training and validation phase, and the remaining 25% for the testing phase. Then, on the first group, stratified tenfold cross-validation was used in each single trial, so as to ensure that all the predictors and the labels are present in each partition of the collected data. This way, each fold is a smaller representation of the whole dataset and possible bias is avoided.

The tested setups for each ML algorithm are shown below.

#### Random forest setups

To train and validate RF algorithm, bootstrap technique was used, by randomly considering one third of the variables at each decision split and repeating for 50 decision trees.

Three options were considered to treat input variables, namely as score (ordinal variables), as numeric values, and as binary values obtained by thresholding the scores and considering a difficulty as present if they are higher than the threshold or not present otherwise. Different thresholds ($Thr$) were tried that is, $Thr=1.5$, $Thr=2.5$, $Thr=3.5$, and $Thr=4.5$. Thus, a total of 6 setups was tested.

#### Regression setups

Since output variables were binarized, logistic regression was used instead of linear regression. The characteristics of the LR algorithm required to consider input variables scores solely as numeric values, thus 1 setup of this algorithm was tested.

#### k-nearest neighbors setups

To train and test kNN algorithm, input variables were treated both as numeric values and as binary value, obtained with the same thresholding method described previously. Euclidean and Hamming distance were used in the first and in the second case, respectively.

The considered values for the k parameter range from 7 to 39, with a step of 4.

A total of 45 different setups (5 options for input variables × 9 values of k) was, thus, considered.

#### Support vector machines setups

Three different kernels were considered in training SVM algorithm that is linear, polynomial and radial basis function (RBF). Some preliminary tests had been carried out earlier to determine which degree of the polynomial kernel allowed the best performance and the result was 2.

Again, input variables were treated both as numeric values and as binary value obtained with the same thresholding method described previously.

Thus, 15 different setups (3 options for kernels × 5 options for input) were tested.

Summing, the tests carried out to find the best classification model for each of the 17 tools and of the 22 strategies were 67. A total of 67 × (17 + 22) = 2613 trials were, thus, performed.

To evaluate the performance of the algorithms, the overall weighted prediction accuracy ($A$) for each tool or strategy was calculated with the following formula:

2 $$A=\frac{1}{{N}_{F}}\cdot \sum_{f=1}^{{N}_{F}}\left(\frac{{N}_{{(C/y)}_{f}}}{{N}_{(T/y)}}\cdot {w}_{y}+\frac{{N}_{{(C/n)}_{f}}}{{N}_{(T/n)}}\cdot {w}_{n}\right) ,$$

where ${N}_{F}$ is the number of folds used for cross-validation (namely 10), ${N}_{{(C/y)}_{f}}$ and ${N}_{{(C/n)}_{f}}$ are the number of correct predictions in the case of “useful” and “useless” algorithm response, respectively, ${N}_{(T/y)}$ and ${N}_{(T/n)}$ are the total number of tests performed in each fold, in the case of “useful” and “useless” response, respectively, and ${w}_{y}$ and ${w}_{n}$ are the weights used to take into consideration the imbalance of the two classes, as previously explained. ${w}_{y}$ and ${w}_{n}$ were set at the normalized inverse frequency of the “useful” and “useless” responses, so as to give a higher importance to less frequent predictions and vice versa. In addition, also F1-score was calculated, so as to include also precision and recall among the performance indexes.

### Final testing of the procedure

The final super-algorithm, composed of the most accurate ML algorithm with its best setup for each single tool and strategy, was then tested on the part of the dataset left for this purpose, by calculating its overall accuracy.

Finally, a further evaluation step was performed in a real scenario. A group of students answered to the questions in Table 1. Their answers were input to the classification model, which output the best support tools and strategies for each of them. Then, the students tried all the suggested tools and strategies and were asked to state which of them were useful in studying and which not. The response of each student was compared with the output of the classification model, to evaluate its accuracy. Note that, among the strategies listed in Table 2b, S10 was not taken into account yet, since it requires a larger time span to be verified. However, a student association have already been created and its verification is ongoing. For the same reason, strategies S11, S17 and S18 were not applied on an entire course, but only on some of its topics. S11 was verified by teaching some topics in class and some other online or only by providing study material (books and notes), without the presence of a teacher. S17, instead, was verified by providing information about specific topics before the beginning of the lessons. Finally, S18 have been tested by grouping topics in shorter sub-topic modules. A total of 102 students having dyslexia participated at this last evaluation step, by answering to the questionnaire. Among its questions, one asks which of the support tools and strategies listed in Table 2 has been extensively or systematically used before. Fifty-six students answered that they have not tried any of them. This group was chosen for the evaluation, since for them no bias should be present. Among them, 13 were not able to participate for personal reasons, whereas 43 took part at the experiment.

## Results

The training and evaluation of the ML algorithms were carried out on the 1217 questionnaire answers that passed the selection procedure exposed in the “Prediction algorithm design” section. As said in the “Preprocessing of the data” section, tool T4 was excluded from the analysis since more than 15% of the participants did not know it. Thus, a total of 16 tools and 22 strategies were effectively included in the analysis.

The weighted prediction accuracy of all the algorithms and their setups, and their F1-score were then calculated and the best performing one for each single tool and strategy was selected. The selection criterium was: “the winner is the algorithm/setup that has the highest mean value of its weighted accuracy and F1-score”. Table 4 reports the names of the most effective algorithms and their best setup, jointly with the achieved prediction accuracy and F1-score, for each tool (a) and strategy (b). The use of these algorithms/setups will constitute the final classification model. Obviously, for the prediction of the i-th tool/strategy, the best performing algorithm/setup for that tool/strategy will be run.

**Table 4:** Best-predicting algorithms and setups for each tool (a) and strategy (b) and accuracy achieved by each of them.

(a)				
Tool	Best performing algorithm	Algorithm setup	Accuracy (weighted)	F1-score
T1	SVM	Linear kernel, binary input (Thr = 1.5)	0.725	0.813
T2	Random forest	Score	0.904	0.940
T3	k-NN	k = 39, numeric input	0.891	0.931
T4	–	–	–	–
T5	SVM	Linear kernel, numeric input	0.836	0.895
T6	Random forest	Score	0.960	0.976
T7	SVM	RBF kernel, numeric input	0.978	0.987
T8	SVM	Linear kernel, numeric input	0.910	0.944
T9	SVM	Linear kernel, numeric input	0.910	0.943
T10	SVM	Linear kernel, numeric input	0.931	0.958
T11	SVM	RBF kernel, numeric input	0.743	0.825
T12	SVM	RBF kernel, numeric input	0.768	0.845
T13	k-NN	k = 31, numeric input	0.949	0.969
T14	SVM	RBF kernel, binary input (Thr = 2.5)	0.967	0.981
T15	SVM	Linear kernel, numeric input	0.913	0.947
T16	SVM	RBF kernel, numeric input	0.897	0.936
T17	k-NN	k = 31, numeric input	0.917	0.949

(b)				
Strategy	Best performing algorithm	Algorithm setup	Accuracy (weighted)	F1-score
S1	SVM	RBF kernel, numeric input	0.705	0.803
S2	k-NN	k = 31, numeric input	0.970	0.982
S3	k-NN	K = 31, numeric input	0.981	0.988
S4	Random forest	Score	0.972	0.984
S5	SVM	RBF kernel, binary input (Thr = 1.5)	0.989	0.994
S6	Random forest	Score	0.980	0.980
S7	k-NN	k = 31, binary input (Thr = 1.5)	0.929	0.957
S8	k-NN	k = 39, numeric input	0.855	0.908
S9	SVM	RBF kernel, numeric input	0.860	0.912
S10	SVM	RBF kernel, binary input (Thr = 1.5)	0.882	0.927
S11	SVM	RBF kernel, numeric input	0.933	0.958
S12	SVM	RBF kernel, numeric input	0.969	0.979
S13	Random forest	Score	0.989	0.994
S14	Random forest	Score	0.994	0.997
S15	k-NN	k = 31, binary input (Thr = 1.5)	0.958	0.975
S16	Random forest	Score	0.971	0.982
S17	k-NN	k = 31, numeric	0.938	0.963
S18	k-NN	k = 31, binary input (Thr = 1.5)	0.971	0.960
S19	SVM	RBF kernel, numeric input	0.768	0.845
S20	SVM	RBF kernel, numeric input	0.790	0.862
S21	SVM	RBF kernel, numeric input	0.825	0.890
S22	SVM	RBF kernel, numeric input	0.931	0.959

The average weighted accuracy achieved globally and singularly for the tools and the strategies is reported in Table 5, jointly with the standard deviation, the minimum and the maximum of the results and the average F1-score.

**Table 5:** Average, standard deviation, maximum and minimum accuracy achieved over all the tools, all the strategies and globally on both.

**Tools**	
Average prediction accuracy	0.887
Accuracy standard deviation	0.079
Maximum accuracy	0.978
Minimum accuracy	0.725
Average F1-score	0.927
**Strategies**	
Average prediction accuracy	0.916
Accuracy standard deviation	0.088
Maximum accuracy	0.994
Minimum accuracy	0.705
Average F1-score	0.945
**Global**	
Average prediction accuracy	0.904
Accuracy standard deviation	0.084
Maximum accuracy	0.994
Minimum accuracy	0.705
Average F1-score	0.938

The weighted accuracy in predicting the tool and the strategies is around 90% in average (88.7% for the former and 91.6% for the latter) and, globally, 90.4% is achieved, with a low standard deviation of 0.079 and 0.088 for tools and strategies, respectively, and 0.084 globally). Thus, the implemented algorithm is capable to output around 9 correct predictions each 10 about the most useful support methodologies for university students with dyslexia, based on the issues they encountered. The good result is confirmed by the F1-score, which is 0.927 for the tools, 0.945 for the strategies and 0.938 in general. The highest accuracy achieved is 0.978 for the tools and 0.994 for the strategies. The lowest one, instead, is 0.725 for the tools and 0.705 for the strategies, which are sensibly worse than the best cases. However, from Table 4, it appears that only 4 tools on 16 and 4 strategies on 22 are predicted with an accuracy lower than 0.85. Thus, in the case that this level of accuracy is considered as not sufficient, it makes sense to renounce to predict the usefulness of these 5 tools/strategies and concentrate on the remaining 30. Table 6 shows how the results changes, with respect to Table 5, if the less predictable tools/strategies are not considered. The average accuracy rises to 92.7% for the tools, 94.8% for the strategies and 94.0% globally, with a standard deviation of 0.029, 0.043 and 0.039 respectively. The minimum accuracy achieved is now 0.855. The average F1-score also rises to 0.955 and 0.968 for tools and strategies, respectively, and to 0.966 globally, giving a further proof of the goodness of the implemented classification model.

**Table 6:** Average, standard deviation, maximum and minimum accuracy achieved over all the tools, all the strategies and globally on both, calculated by excluding predictions with a weighted accuracy lower than 85%.

**Tools (performances < 85% discarded)**	
Average prediction accuracy	0.927
Accuracy standard deviation	0.029
Maximum accuracy	0.978
Minimum accuracy	0.891
Average F1-score	0.955
**Strategies (performances < 85% discarded)**	
Average prediction accuracy	0.948
Accuracy standard deviation	0.043
Maximum accuracy	0.994
Minimum accuracy	0.855
Average F1-score	0.968
**Global (performances < 85% discarded)**	
Average prediction accuracy	0.940
Accuracy standard deviation	0.039
Maximum accuracy	0.994
Minimum accuracy	0.855
Average F1-score	0.963

It is worth noting that, even choosing the best performing ML algorithm with its best setup among all the tested ones, the achieved performance is considerably lower than by choosing a different algorithm for the prediction of each tool and strategy. In particular, SVM with numeric input and RBF kernel, which reached the highest average weighted accuracy (86.5% and 89.4%, by considering and discarding the performances under 85% accuracy, respectively), showed a performance around 4% lower than the implemented method. This justifies the choice made.

It is however interesting to compare the single algorithms performance. From Table 4 it can be noted that, as said, SVM is the algorithm that most of the times (21 on 38) outperforms the others, especially when the input is considered as numeric and RBF kernel is used. k-NN follows (10 times on 38), with k set at 31 (8 times) and 39 (twice) and, mostly, with numeric input. Then, random forest wins 7 times on 38, always when the input is considered as a score. Logistic regression never outperforms the other algorithms.

Once the best prediction algorithm had been determined and implemented, it was tested on a real case. As said, the test consisted in comparing the support tools and strategies that a sample of 43 dyslexic students found useful or useless, after trying them in studying, with the prediction output by the algorithm when fed with the issues experienced by the students during their career. The results, reported in Table 7, confirmed that the proposed prediction algorithm performs very accurately. Among all the support methodologies (tools plus strategies) predicted as useful, more than 92% are actually useful and, among all those predicted as useless, almost 90% are actually so. Similar results were obtained for tools and strategies singularly. This demonstrate that the algorithm can be profitably employed to predict the best support methodologies for dyslexic students.

**Table 7:** Prediction accuracy of the proposed algorithm, calculated on the tested real case.

	Actually useful (as stated by students)	Actually useless (as stated by students)
Predicted as useful (by the proposed algorithm)	Tools: 90.3%	Tools: 9.7%
	Strategies: 94.1%	Strategies: 5.9%
	Global: 92.4%	Global: 7.6%
Predicted as useless (by the proposed algorithm)	Tools: 13.4%	Tools: 86.6%
	Strategies: 8.7%	Strategies: 91.3%
	Global: 10.7%	Global: 89.3%

*Percentages refer to the actually useful/useless support methodologies on the total of useful/useless predictions.*

## Conclusions

This paper deals with the possibility to offer customized support to university students with dyslexia, by creating a classification model of the most useful digital tools and learning strategies for each of them singularly, based on the issues they have generally encountered during their educational journey.

To do this, an AI algorithm has been implemented, which is based on effective ML techniques at the state of the art. In particular, four ML algorithms with different setups have been trained and tested and the most performing combination in predicting each tool/strategy has been chosen to predict that tool/strategy. To collect the data needed for the algorithm training/testing, a questionnaire about the difficulties encountered while studying and the most helpful support methodologies has been created and then spread to dyslexic university students. Questionnaire items must be answered with a Likert scale-based level of satisfaction, which was converted to a score between 0 and 5 and then thresholded at half of the score range, in the case of support methodologies, to obtain a yes/no response to their utility. The thresholding operation could introduce possible misclassifications but, for the scope of this work, it is a step that must be performed. Indeed, it is of primary importance to suggest clearly to students with dyslexia if each tool/strategy can be useful for their or not, avoiding the noise that can arise from the possible misunderstanding of the meaning of a utility score.

The results of the testing of the final algorithm show that the prediction accuracy, which was opportunely weighted to take into account a class imbalance of the considered variables, ranges from 72.5 to 97.8% for the tools and from 70.5 to 99.4% for the strategies, with an average of 88.7% and 91.6%, respectively. At a global level, the achieved average accuracy is 90.4%. In addition, the low standard deviation suggests that the prediction accuracy is around the average for the majority of the tools and strategies. Thus, it is meaningful to state that the implemented algorithm is capable of predicting correctly, in around 9 cases each 10. Its precision is also confirmed, by the high F1-score (0.927 for the tools, 0.945 for the strategies and 0.938 globally).

Average prediction accuracy has been also recalculated after discarding those tools and strategies for which a value lower than 85% had been achieved, which are only 4 per category. In this case, the average accuracy rises to 92.7% for the tools, 94.8% for the strategies and 94.0% globally. In addition, the proposed algorithm has been tested on a real case, achieving a prediction accuracy higher than 90% in suggesting useful and useless support methodologies to a sample of 43 dyslexic students.

Comparisons with other approaches could not be made, since, to the best of authors’ knowledge after a thorough literature review, this is the first approach that aims at applying AI to estimate the most proper support methodologies in a personalized way for each student. Indeed, AI has been generally used directly to create aid tools, regardless the student-specific needs^19–21^, or as a diagnostic tool^10–14^. The only study focused on the selection of personalized learning experience based on different dyslexia types is the one proposed in^22^. However, a validation procedure of the used algorithms has not been reported and prediction accuracy has not been calculated. Furthermore, due to the absence of complete clinical reports of dyslexia made by experts, also a comparison between the tools and strategies suggested by the implemented algorithm and the ones suggested by the experts could not have been made. This represents a limitation in the validation procedure. Another limitation lies in the fact that the evaluation of the accuracy of the algorithm have been performed only in the short and medium term. A long-term evaluation process is needed and is being performing.

Despite of these limitation, the obtained results prove that the implemented classification model can be successfully used to provide dyslexic students at university with personalized support tools and strategies, given the issues that have affected their learning path. This achievement opens the door to a new way of thinking and acting within university institutions about the problem of dyslexia, which aims at boosting the inclusivity by changing the teaching modalities toward the affected students’ needs, thanks to the use of the new digital techniques and technologies, instead of simply decreasing the study load, giving more time to prepare exams, cutting some exams from the programme etc., as it has been done until now.

This work will follow as provided by the objectives of the above-mentioned VRAIlexia project (“Introduction” section) that is, the predicted tools and strategies for each student will be tested further by themselves, so as to obtain feedback about their usefulness. Through such feedback, tools and strategies will be refined to meet the students’ requirements and again tested by them. This process will be repeated until obtaining support tools and strategies which are fully personalized. Reinforcement learning techniques will be explored to try to reach the goal. At the finish line, a considerable step toward a real and fair inclusion within university of all the students will have been taken.

## Data Availability

The dataset generated and analysed during the current study is not publicly available, since it is protected by the GDPR 2016/679. It can be made available from the corresponding author on reasonable request, to be forwarded to a specific panel of the European Union, which must give its approval.
